# Magnetic resonance imaging study in a normal Bengal tiger (*Panthera tigris*) stifle joint

**DOI:** 10.1186/s12917-015-0532-4

**Published:** 2015-08-11

**Authors:** Alberto Arencibia, Mario Encinoso, José R. Jáber, Daniel Morales, Diego Blanco, Alejandro Artiles, José M. Vázquez

**Affiliations:** Department of Morphology, Veterinary Faculty, University of Las Palmas de Gran Canaria, Trasmontaña s/n, 35413 Arucas, Gran Canaria Spain; Veterinary Hospital Los Tarahales, Recta de Los Tarahales 15, 35013 Las Palmas de Gran Canaria, Spain; Department of Anatomy and Comparative Anatomy, Veterinary Faculty, University of Murcia, Campus de Espinardo, 30071 Murcia, Spain

**Keywords:** Magnetic resonance imaging, Anatomy, Stifle joint, Tiger

## Abstract

**Background:**

The purpose of this study was to describe the normal appearance of the bony and soft tissue structures of the stifle joint of a Bengal tiger (*Panthera tigris*) by low-field magnetic resonance imaging (MRI), and the use of gross anatomical dissections performed as anatomical reference. A cadaver of a mature female was imaged by MRI using specific sequences as the Spin-echo (SE) T1-weighting and Gradient-echo (GE) STIR T2-weighting sequences in sagittal, dorsal and transverse planes, with a magnet of 0.2 Tesla. The bony and articular structures were identified and labelled on anatomical dissections, as well as on the magnetic resonance (MR) images.

**Results:**

MR images showed the bone, articular cartilage, menisci and ligaments of the normal tiger stifle. SE T1-weighted sequence provided excellent resolution of the subchondral bones of the femur, tibia and patella compared with the GE STIR T2-weighted MR images. Articular cartilage and synovial fluid were visualised with high signal intensity in GE STIR T2-weighted sequence, compared with SE T1-weighted sequence where they appeared with intermediate intensity signal. Menisci and ligaments of the stifle joint were visible with low signal intensity in both sequences. The infrapatellar fat pad was hyperintense on SE T1-weighted images and showed low signal intensity on GE STIR T2-weighted images.

**Conclusions:**

MRI provided adequate information of the bony and soft tissues structures of Bengal tiger stifle joints. This information can be used as initial anatomic reference for interpretation of MR stifle images and to assist in the diagnosis of diseases of this region.

## Background

In the Felidae family (subfamily pantherinae), six species of tigers belonging to the genus Panthera have been recognized [1]. Since 2010, it has been classified as an endangered species by the International Union for Conservation of Nature and Natural Resources [2]. Many veterinarians and wildlife researchers are involved in large non-domestic feline conservation in zoo and wildlife rehabilitation centers. Conservation task includes anatomical, physiological and clinical works [3–5]. However, studies by means of imaging exploratory techniques have been very sparse and they included radiographic and computed tomography (CT) for anatomical evaluation of the tiger stifle [6, 7], the assessing of a fracture of tibia and fibula [8], and different head diseases such as a nasopharyngeal myxosarcoma [9], an extradural hematoma [10], and a retrobulbar abscess [11].

In relation to magnetic resonance imaging (MRI), the studies done in this species have been limited to the study of central infarction and haemorrhage [12] and suspected neurotoxicity [13], but to date, no detailed MRI anatomic studies of stifle joints in large non-domestic cat have been reported. However, MRI has been used for descriptive anatomic researches of the stifle joints in dog [14–17] and horse [18], as well as to demonstrate the clinical value of MRI in diagnosing diseases of this region [19–23]. To the author’s knowledge, the lack of MRI stifle studies performed in domestic cats could be due to the small size of the feline stifle joint structures that require the use of high field MRI equipment to obtain high definition images.

The stifle joint is one of the more complexes of the locomotor system and it is particularly interesting because their numerous anatomic structures such as the bones, articular cartilage, menisci and ligaments. Knowledge of feline-specific anatomy is important to avoid over- or misinterpretation of physical examination or imaging findings [24]. Therefore, an accurate interpretation of MRI anatomy of the stifle would be useful in diagnosis of diseases described in feline medicine [25–29]. The aim of this study was to describe the normal anatomy of the stifle joint of a Bengal tiger (Panthera tigris), using gross anatomical dissections, and sagittal, dorsal and transverse MR images.

## Methods

### Animals

A cadaver of 6-year-old female Bengal tiger (*Panthera tigris*) with a weight of 105 kg, born in captivity in Cocodrilos Park Zoo (Gran Canaria, Canary Islands, Spain) that died for natural causes not related to stifle joint disorder was used. The experimental study was conducted with authorization of the Conservation Nature Service (Seprona) of Gran Canaria at the Spanish Ministry of Interior (Protocol 2012) and the control of the Ethical Commission of Veterinary Medicine of the University of Las Palmas de Gran Canaria (agreement MV-2015/05).

### MRI technique

After her death, the tiger was immediately frozen. Two days later, the animal was defrosted during 48 h to perform the MR imaging study using a 0.2-Tesla magnet (Vet-MR Esaote, Genova, Italy). The animal was positioned in right lateral recumbency on the scanning table with the hindlimbs in semi-extended position. Each stifle joint was subjected to MRI examination. Spin-echo (SE) T1-weighted and Gradient-Echo (GE) STIR T2-weighted MR images were obtained in 2D sagittal, dorsal and tranverse planes. SE T1-weighted sagittal images were acquired with the following settings: echo time (TE) = 26 msec, repetition time (TR) = 760 msec, number of acquisitions (NEX) = 3, 4 mm slice thickness with 4.4 mm spacing between slices, a field of view of 512 × 512 and a matrix of 192 × 136. For GE STIR T2-weighted sagittal images, the TE was 25 msec, TR was 1500 msec, inversion time (TI) was 75 msec, flip angle was 90°, number of acquisitions (NEX) = 2, 4 mm slice thickness with 4.4 mm interslice spacing, a field of view of 256 × 256 and a matrix of 192 × 136 matrix. For SE T1-weighted dorsal images, the TE was 26 msec, TR was 690 msec, number of acquisitions (NEX) = 3, and 4.5 mm slice thickness with 4.9 mm interslice spacing, a field of view of 256 × 256 and a matrix of 256 × 172. For GE STIR T2-weighted dorsal images, the TE was 25 msec, TR was 1680 msec, TI was 75 msec, flip angle was 90°, number of acquisitions (NEX) = 2, 4.5 mm slice thickness with 4.9 mm interslice spacing, a field of view of 256 × 256 and a matrix of 192 × 136. For SE T1-weighted transverse images, the TE was 26 msec, TR was 640 msec, number of acquisitions (NEX) = 3, 4 mm slice thickness with 4.4 mm interslice spacing, a field of view of 512 × 512 and a matrix of 288 × 199. For GE STIR T2-weighted transverse images, the TE was 25 msec, TR was 1600 msec, TI was 75 msec, flip angle was 90°, number of acquisitions (NEX) = 2, 4 mm slice thickness with 4.4 mm interslice spacing, a field of view of 256 × 256 and a matrix of 192 × 136.

### Anatomic evaluation

At the conclusion of the MR study, gross anatomical dissections of the stifle joint, osseous preparations and textbooks of anatomy [30–33] were used to facilitate an accurate interpretation of the MR images.

## Results

**Fig. 1:**
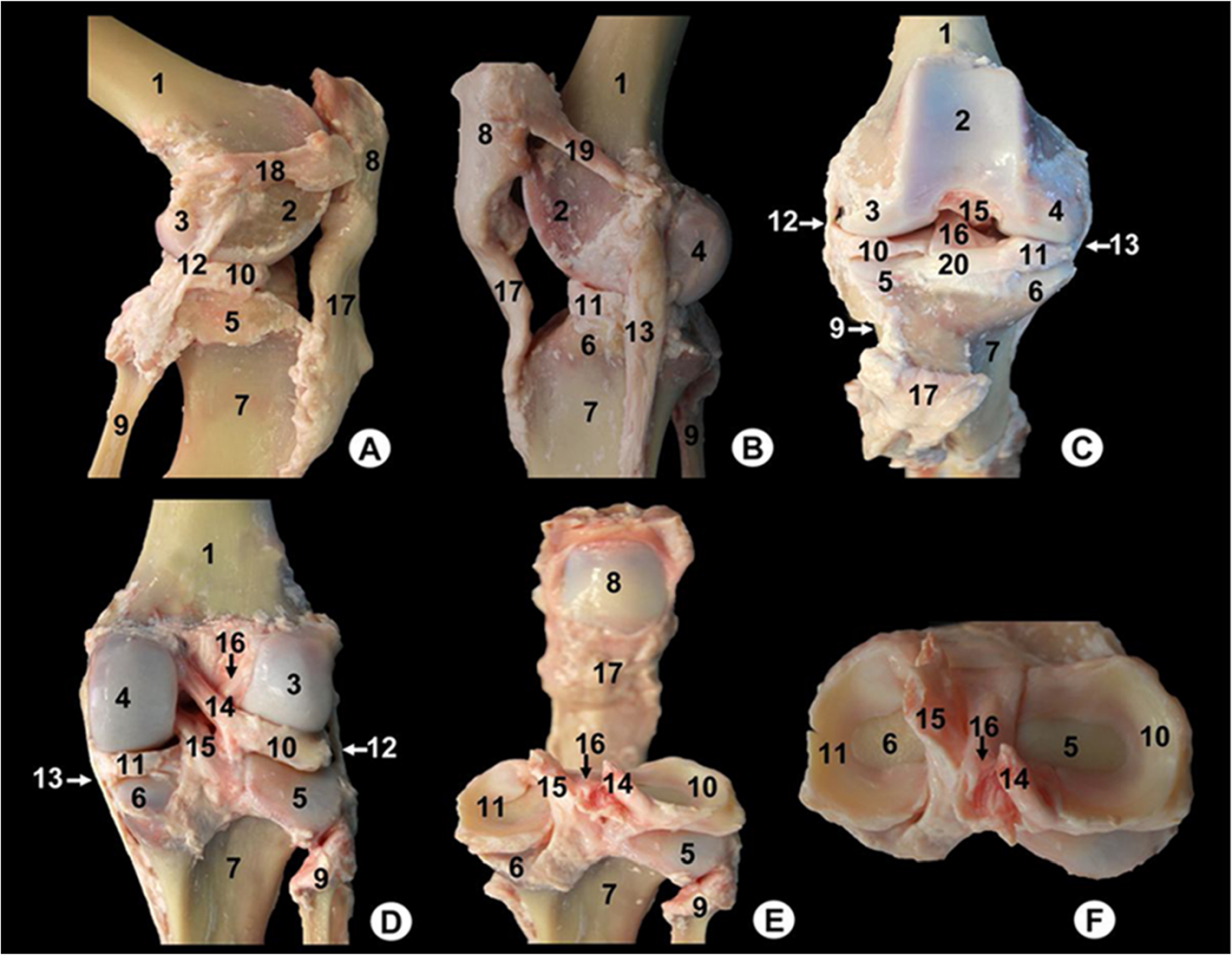
Gross anatomical dissections of Bengal tiger stifle joint. **a** lateral aspect, **b** medial aspect, **c** cranial aspect with joint opened, **d** caudal aspect, **e** caudal aspect with the femur removed and **f** proximal aspect of the meniscus and cruciate ligaments. 1. Femoral metaphysis, 2. Femoral trochlea, 3. Lateral femoral condyle, 4. Medial femoral condyle, 5. Lateral tibial condyle, 6. Medial tibial condyle, 7. Tibial metaphysis, 8. Patella, 9. Fibula, 10. Lateral meniscus, 11. Medial meniscus, 12. Lateral collateral ligament, 13. Medial collateral ligament, 14. Meniscofemoral ligament, 15. Caudal cruciate ligament, 16. Cranial cruciate ligament, 17. Patellar ligament, 18. Lateral patellofemoral ligament, 19. Medial patellofemoral ligament, 20. Transverse ligament of stifle

**Fig. 2:**
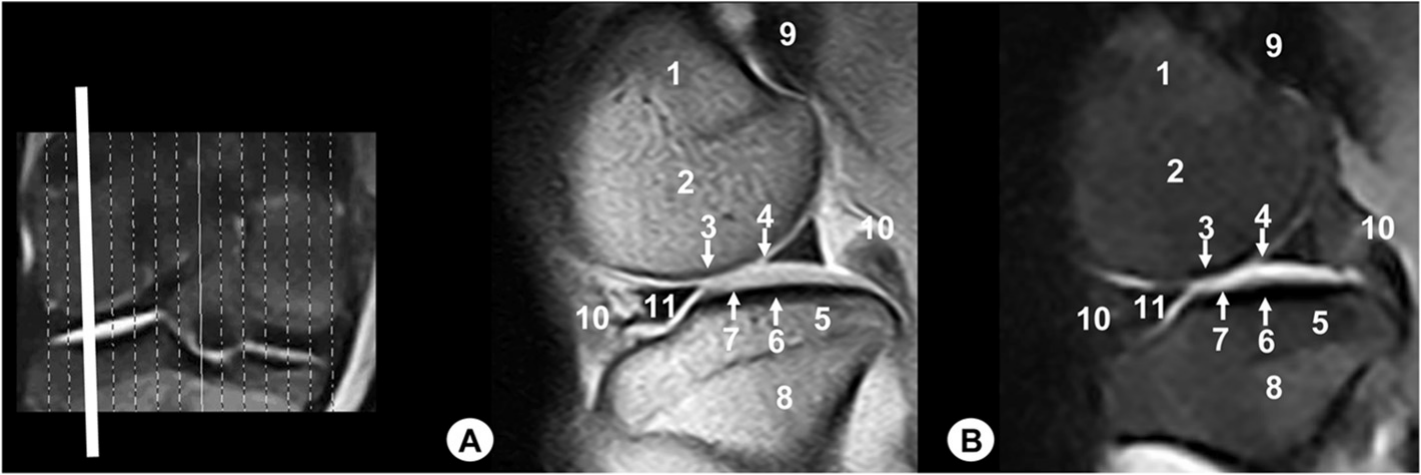
Sagittal MR images of stifle joint. Lateral view. Line depicts the section at the level of the lateral femoral condyle. **a** SE T1-weighted MR image and **b** GE STIR T2-weighted MR image. 1. Femoral metaphysis, 2. Lateral femoral condyle, 3. Cortical bone of the femur, 4. Articular cartilage of the femur, 5. Lateral tibial condyle, 6. Cortical bone of the tibia, 7. Articular cartilage of the tibia, 8. Tibial metaphysis, 9. Lateral sesamoid bone of the gastrocnemius muscle, 10. Femorotibial joint (articular capsule), 11. Lateral meniscus

**Fig. 3:**
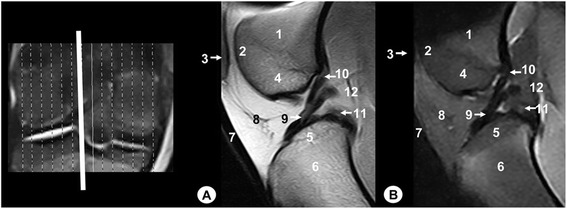
Sagittal MR images of stifle joint. Lateral view. Line depicts the section at the level of the lateral surface of the intercondylar notch of the femur. **a** SE T1-weighted MR image and **b** GE STIR T2-weighted MR image. 1. Femoral metaphysis, 2. Femoral trochlea, 3. Patella, 4. Lateral femoral condyle, 5. Lateral tibial condyle, 6. Tibial metaphysis, 7. Patellar ligament, 8. Infrapatellar fat pad, 9. Cranial cruciate ligament, 10. Meniscofemoral ligament, 11. Caudal cruciate ligament, 12. Femorotibial joint (articular capsule)

**Fig. 4:**
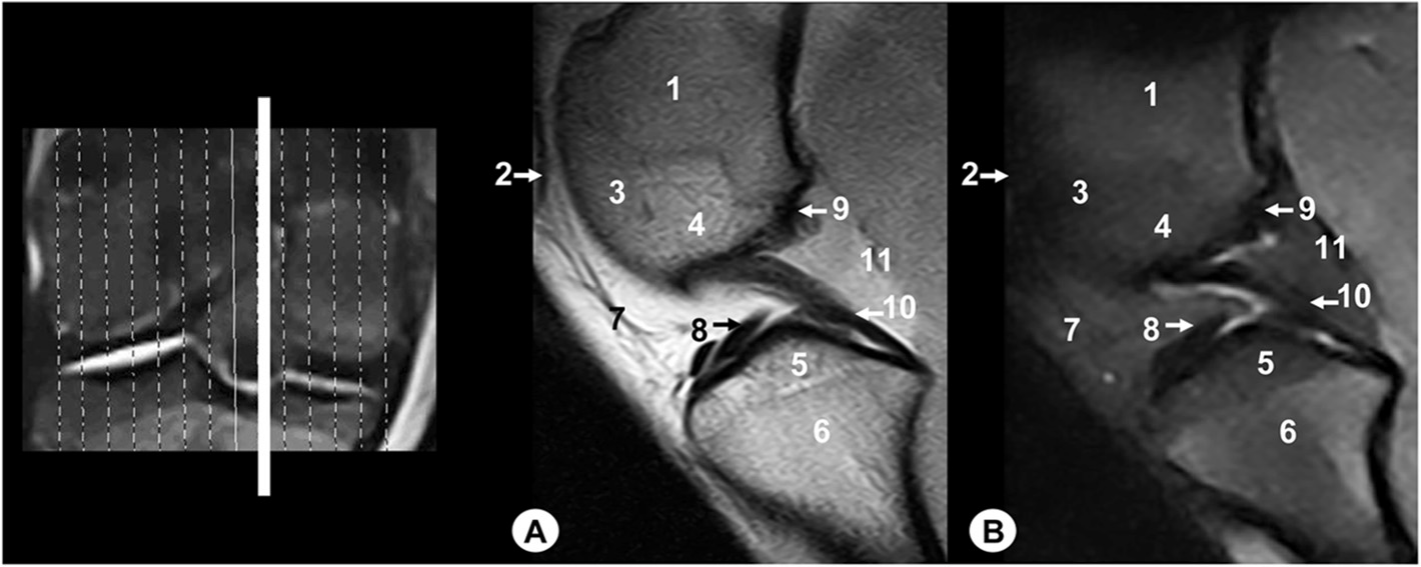
Sagittal MR images of stifle joint. Lateral view. Line depicts the section at the level of the medial surface of the intercondylar notch of the femur. **a** SE T1-weighted MR image and **b** GE STIR T2-weighted MR image. 1. Femoral metaphysis, 2. Patella, 3. Femoral trochlea, 4. Medial femoral condyle, 5. Medial tibial condyle, 6. Tibial metaphysis, 7. Infrapatellar fat pad, 8. Cranial cruciate ligament, 9. Meniscofemoral ligament, 10. Caudal cruciate ligament, 11. Femorotibial joint (articular capsule)

**Fig. 5:**
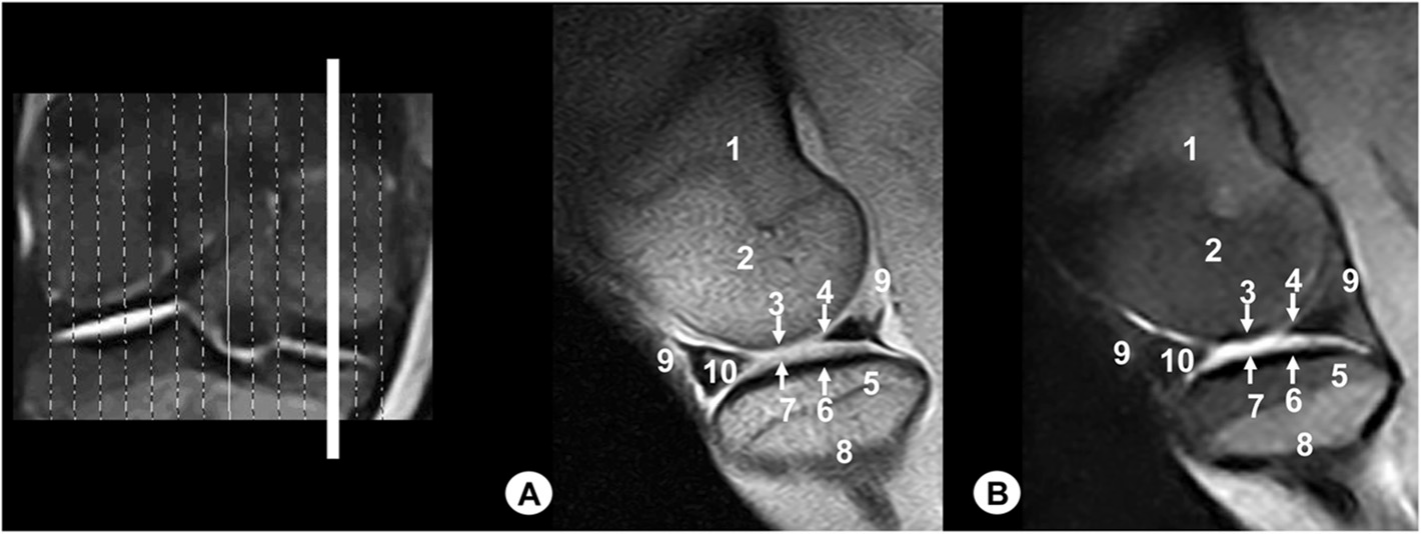
Sagittal MR images of stifle joint. Lateral view. Line depicts the section at the level of the medial femoral condyle. **a** SE T1-weighted MR image and **b** GE STIR T2-weighted MR image. 1. Femoral metaphysis, 2. Medial femoral condyle, 3. Cortical bone of the femur, 4. Articular cartilage of the femur, 5. Medial tibial condyle, 6. Cortical bone of the tibia, 7. Articular cartilage of the tibia, 8. Tibial metaphysis, 9. Femorotibial joint (articular capsule), 10. Medial meniscus

**Fig. 6:**
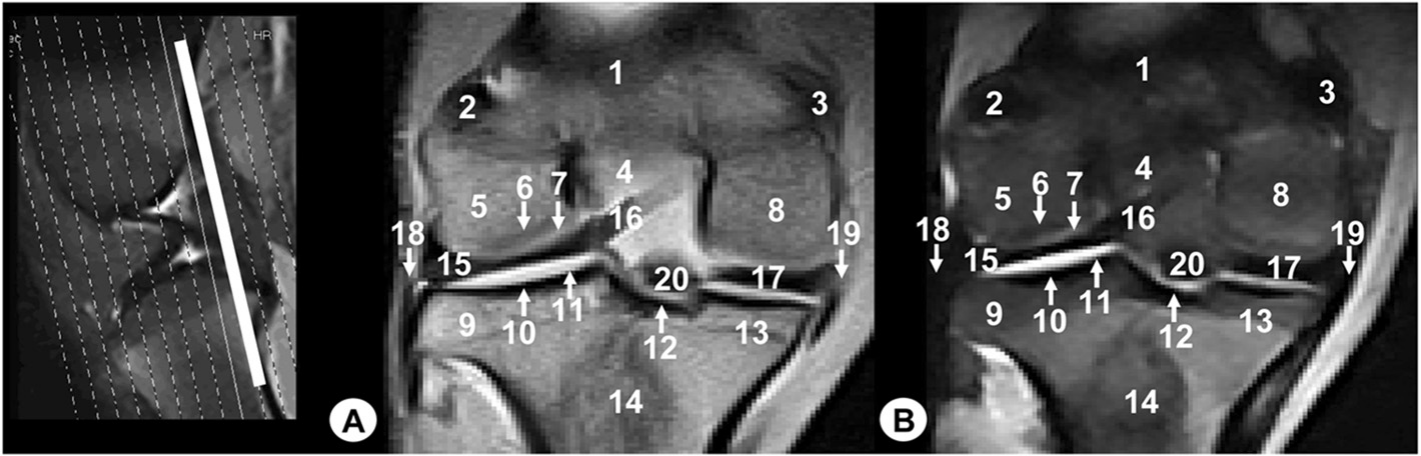
Dorsal MR images of stifle joint. Cranial view. Line depicts the level of section at the level of the meniscofemoral ligament. **a** SE T1-weighted MR image and **b** GE STIR T2-weighted MR image. 1. Femoral metaphysis, 2. Lateral sesamoid bone of the gastrocnemius muscle, 3. Medial sesamoid bone of the gastrocnemius muscle, 4. Intercondylar notch of the femur, 5. Lateral femoral condyle, 6. Cortical bone of the femur, 7. Articular cartilage of the femur, 8. Medial femoral condyle, 9. Lateral tibial condyle, 10. Cortical bone of the tibia, 11. Articular cartilage of the tibia, 12. Caudal intercondylar area of the tibia, 13. Medial tibial condyle, 14. Tibial metaphysis, 15. Lateral meniscus, 16. Meniscofemoral ligament, 17. Medial meniscus, 18. Lateral collateral ligament, 19. Medial collateral ligament, 20. Caudal cruciate ligament

**Fig. 7:**
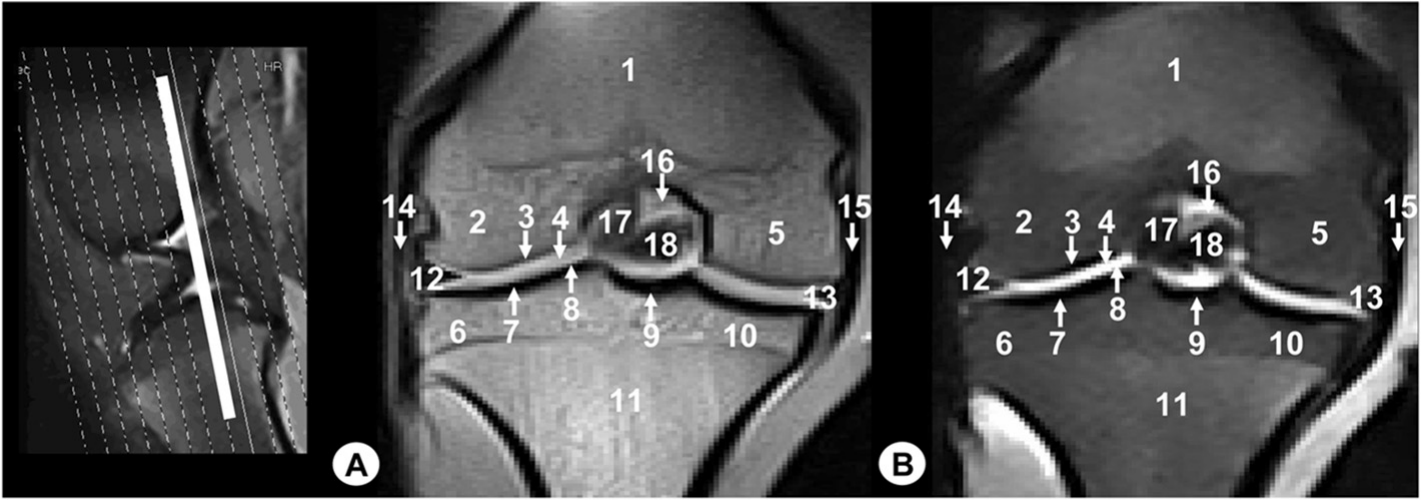
Dorsal MR images of stifle joint. Cranial view. Line depicts the section at the level of the cruciate ligaments of stifle. **a** SE T1-weighted MR image and **b** GE STIR T2-weighted MR image. Cranial view. 1. Femoral metaphysis, 2. Lateral femoral condyle, 3. Cortical bone of the femur, 4. Articular cartilage of the femur, 5. Medial femoral condyle, 6. Lateral tibial condyle, 7. Cortical bone of the tibia, 8. Articular cartilage of the tibia, 9. Central intercondylar area of the tibia, 10. Medial tibial condyle, 11. Tibial metaphysis, 12. Lateral meniscus, 13. Medial meniscus, 14. Lateral collateral ligament, 15. Medial collateral ligament, 16. Meniscofemoral ligament, 17. Cranial cruciate ligament, 18. Caudal cruciate ligament

**Fig. 8:**
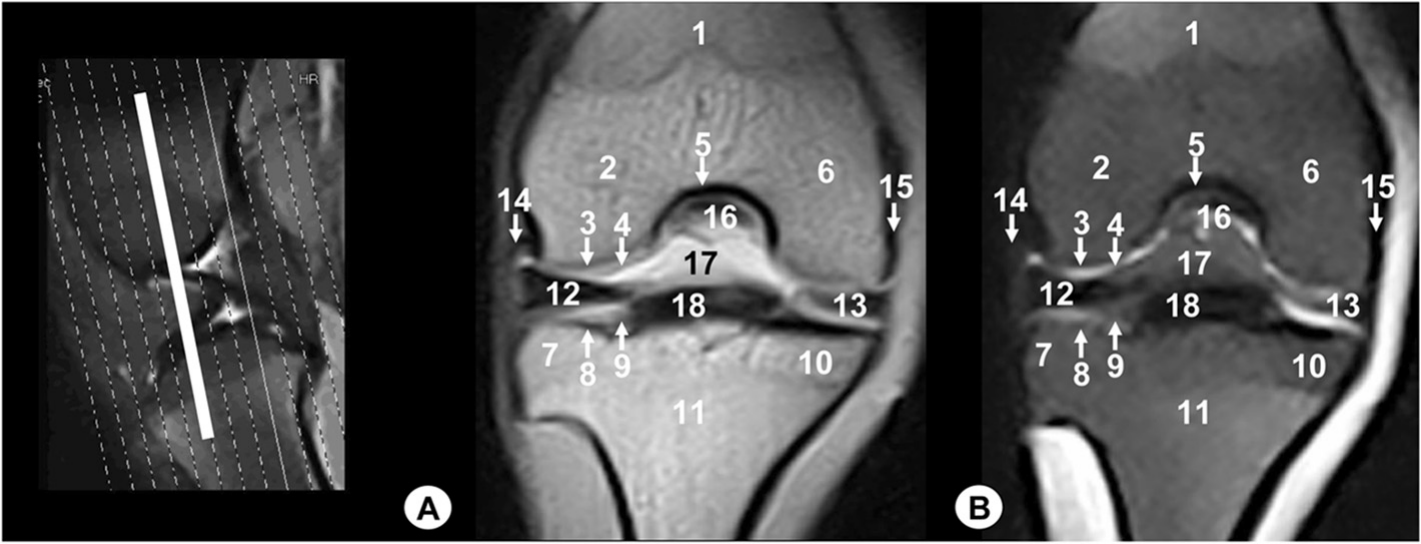
Dorsal MR images of stifle joint. Cranial view. Line depicts the section at the level of the transverse ligament of stifle. **a** SE T1-weighted MR image and **b** GE STIR T2-weighted MR image. 1. Femoral metaphysis, 2. Lateral femoral condyle, 3. Cortical bone of the femur, 4. Articular cartilage of the femur, 5. Intercondylar notch of femur, 6. Medial femoral condyle, 7. Lateral tibial condyle, 8. Cortical bone of the tibia, 9. Articular cartilage of the tibia, 10. Medial tibial condyle, 11. Tibial metaphysis, 12. Lateral meniscus, 13. Medial meniscus, 14. Lateral collateral ligament, 15. Medial collateral ligament, 16. Cranial cruciate ligament, 17. Femorotibial joint (articular capsule), 18. Transverse ligament of stifle

**Fig. 9:**
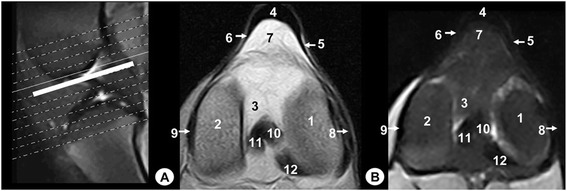
Transverse MR images of stifle joint. Dorsal view. Line depicts the section at the level of cruciate ligaments of stifle. **a** SE T1-weighted MR image and **b** GE STIR T2-weighted MR image. Views are dorsal. 1. Lateral femoral condyle, 2. Medial femoral condyle, 3. Intercondylar notch of the femur, 4. Patellar ligament, 5. Lateral patellofemoral ligament, 6. Medial patellofemoral ligament, 7. Infrapatellar fat pad, 8. Lateral collateral ligament, 9. Medial collateral ligament, 10. Cranial cruciate ligament, 11. Caudal cruciate ligament. 12. Meniscofemoral ligament

**Fig. 10:**
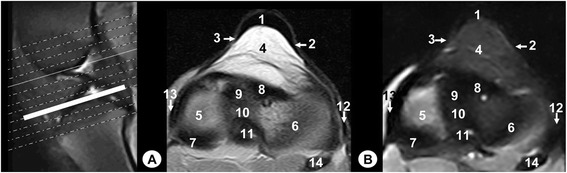
Transverse MR images of stifle joint. Dorsal view. Line depict the section at the level of the menisci and transverse ligament of stifle. **a** SE T1-weighted MR image and **b** GE STIR T2- weighted MR image. Views are dorsal. 1. Patellar ligament, 2. Lateral patellofemoral ligament, 3. Medial patellofemoral ligament, 4. Infrapatellar fat pad, 5. Articular cartilage of the tibia, 6. Lateral meniscus, 7. Medial meniscus, 8. Transverse ligament of stifle, 9. Cranial intercondylar area of the tibia, 10. Central intercondylar area of the tibia, 11. Caudal intercondylar area of the tibia, 12. Lateral collateral ligament, 13. Medial collateral ligament, 14. Fibula

The main structures were labelled on gross anatomical dissections and MRI images. Gross anatomical dissections of the tiger stifle joint from different views are presented in Fig. 1. Nine representative MR images in different planes were selected. Therefore, the sagittal MR images are shown in a lateromedial direction from the lateral to the medial femoral condyles (Figs. 2, 3, 4 and 5). The dorsal MR images began at the level of the caudal intercondylar area of the tibia and continued cranially to the transverse ligament of the stifle (Figs. 6, 7 and 8). The transverse MR images are shown in a proximodistal direction from the cruciate ligaments of stifle to the menisci and transverse ligament of the stifle (Figs. 9 and 10).

On the gross anatomical dissections, the femur (including the metaphysis, the trochlea, the lateral and medial condyles and the intercondylar notch), the tibia (with their metaphysis, the lateral and medial condyles, and the intercondylar eminence), the patella and the head of fibula could be identified (Fig. 1). The articular structures of the femorotibial joint such as the lateral meniscus with its meniscofemoral ligament attached proximally to the inner surface of the medial femoral condyle (Fig. 1d-f), as well as the transverse ligament of stifle that connects the menisci cranially (Fig. 1c) were observed. The cruciate ligaments of stifle were also visible, especially in the cranial (Fig. 1c), caudal (Fig. 1d, e) and dorsal views (Fig. 1f). Therefore, the cranial cruciate ligament, coursing from the inner surface of lateral femoral condyle to the central intercondylar area of the tibia was seen. In contrast, the caudal cruciate ligament was observed from the inner surface of medial femoral condyle to the caudal intercondylar area of the tibia. In relation to the lateral and medial collateral ligaments, they were seen from the lateral femoral epicondyle to the head of fibula and tibia, and from the medial femoral epicondyle to the tibia, respectively (Fig. 1a-d).

In addittion, several articular structures of the femoropatellar joint were identified. The patellar ligament that connected the distal tip of the patella to the tibial tuberosity was seen (Fig. 1a, b). The lateral and medial femoropatellar ligaments were extended from the lateral border of the patella to the lateral sesamoid bone of the gastrocnemius muscle (Fig. 1a), and from the medial border of the patella to the medial sesamoid bone of gastrocnemius muscle (Fig. 1b), respectively.

On the MR images (Figs. 2, 3, 4, 5, 6, 7, 8, 9 and 10), anatomic details of Bengal tiger stifle joints were compared with the anatomical dissections and evaluated according to the signal intensity of the different bony components and soft-tissues structures. Several structures of femorotibial joint were clearly visible with low-field MRI. Therefore, on SE T1-weighted sequences, the signal intensity of the cortical and subchondral bone of the femur and tibia was lower compared with the high signal intensity of the trabecular bone. However, on GE STIR T2-weighted MR images, the trabecular bone appeared with low signal intensity, as well as by observing the area of negligible signal corresponding to the cortical and subchondral bone of those bones. The articular cartilage of the femur and tibia was visualised with intermediate signal intensity in SE T1-weighted sequences, compared with GE STIR T2-weighted sequences where they showed high intensity signal. In both sequences, the signal of the articular cartilage was differentiated from trabecular bone by a dark line that corresponded to the subchondral bone (Figs. 2, 5, 6, 7 and 8). Articular capsule, menisci and ligaments were also seen with low signal intensity in both sequences. The synovial fluid could be identified in the articular cavity with intermediate signal intensity on the T1-weighted MR images. However, this fluid showed high signal intensity in GE STIR T2-weighted sequences.

The lateral and medial menisci were easily identified between the femoral condyles and the tibia as two triangular formations. These structures had low signal intensity in the sagittal (Figs. 2 and 5) and dorsal (Figs. 6, 7 and 8) planes, but they were not clearly observed in the transverse plane (Fig. 10). The meniscofemoral ligament was visible on the sagittal (Figs. 3 and 4), dorsal (Figs. 6 and 7) and transverse (Fig. 9) planes with low signal intensity. The cruciate ligaments were not completely distinguished in all three planes because of their oblique orientation and appeared with low signal intensity (Figs. 3, 4, 7 and 9). The lateral and medial collateral ligaments were well defined on the dorsal (Figs. 6, 7 and 8) and transverse (Figs. 9 and 10) planes as linear low signal intensity bands. These structures were difficult to differentiate to the cortical bone because of their similar signal intensity.

Several structures of femoropatellar joint were also seen with MRI. The patella was well visualized on SE T1-weighted sequences compared with GE STIR T2-weighted MR images, especially on the sagittal (Figs. 3 and 4) and transverse (Fig. 9) planes. On SE T1-weighted images, the patella showed intermediate signal intensity due to the trabecular bone, although the cortical bone appeared with low intensity signal on SE T1-weighted images. However, on GE STIR T2-weighted images, the cortical and trabecular bone of the patella had lower signal intensity. The patellar ligament (Figs. 3, 9 and 10) and the lateral and medial femoropatellar ligaments (Figs. 9 and 10) were well defined (Figs. 9 and 10) and appeared with low signal intensity in both sequences. The infrapatellar fat pad had high signal intensity on SE T1-weighted images and low signal intensity on GE STIR T2-weighted sequences.

## Discussion

Exploration and clinical evaluation of the bony and soft tissue structures of the stifle joint of Bengal tiger is laborious because of their anatomical complexity that makes difficult to diagnose morphological changes only by means of physical examination. In feline medicine, the patient has an excellent prognosis for return to normal stifle function with all but the most severe knee injuries. Thorough physical and radiographic evaluation of the stifle predicts successful management of stifle injury [26].

Diagnostic imaging techniques such as radiography, ultrasonography, CT and MRI have been used to evaluate the bony and soft-tissue structures of the stifle joint [15]. In some cases, radiography may be sufficient to make a diagnosis when the clinical signs and physical examination give full information of the disorder [8, 34]; however, if radiographic assessment is inconclusive, more advanced imaging techniques are necessary [35]. Ultrasonography is considered helpful to evaluate menisci, ligaments and tendons, although the animal’s size can limit their visualization [15]. In contrast, CT has shown to be particularly beneficial for evaluating stifle osseous structures [7, 36], whereas the use of other modalities of this technique as CT arthrography may identify abnormalities of the cruciate ligaments, but is of questionable value for assessing the menisci [37]. However, MR imaging is highly recommended for visualization of the normal stifle joint [14–18] and become the preferred imaging method for the evaluation of diseases in the articular cartilage, menisci and ligaments of synovial joints [19–23].

In veterinary medicine, different magnetic fields scanners have been used to the study of the stifle joint. Some works used a high field magnet that provided excellent resolution of the anatomic structures of the equine stifle joint [17]. In the present study, the images were obtained using a low-field MRI magnet that showed adequate visualization of the normal structures of the tiger stifle joint. Similar results have been observed in dogs using low field intensity protocol [14–16, 18]. The main limitations of low-field MR imaging result from a lower signal-to-noise ratio, poor spatial resolution and motion artefacts are compensated by increasing the section thickness; the reduction in-plane resolution; the increase of the number of acquisitions and consecutively the acquisition time; and decreasing the bandwidth. In addition, with recent improvements, as a better homogeneity of magnets, better technology of the receiver coils and specialized pulse sequences have renewed interest in low-field MRI [38].

In our study, the SE T1-weighted and the GE STIR T2-weighted sequences were selected. Similar sequences have been used in other studies of the normal stifle in the dog [15, 16]. In contrast, Baird et al. [14] and Holcombe et al. [18] used only the SE T1 weighted sequence in the canine and the equine stifle studies, respectively. Recently, other authors have used the fast spin echo T2-weighted sequence to analyse the effects of stifle flexion angle in the evaluation of the normal canine cranial cruciate ligament [18].

SE T1-weighted and GE STIR T2-weighted MR images provided excellent detail of bone, soft-tissues, and synovial fluid of tiger stifle joint. However, SE T1-weighted sequences gave a better definition of the anatomic structures of the femorotibial and femoropatellar joints compared to GE STIR T2-weighted sequences.

As in other works done in dogs [15, 16] and horses [17], all the Bengal tiger stifle joint structures were showed in sagittal, dorsal and transverse MRI planes. However, in studies performed in normal canine stifle joint [14] that only use the sagittal and dorsal MRI planes, specific structures such as the femoropatellar ligaments were not visible.

In the present study, the articular cartilage of the femur and tibia, the cruciate and the meniscofemoral ligaments were visualized in the three anatomic planes. However, the articular cartilage was not clearly defined because the presence of synovial fluid that showed similar intensity signal. Menisci were visualized in all three planes, especially in the sagittal and dorsal planes due to a better identification of their shape. Gross anatomical dissections and MRI examinations revealed meniscal mineralization in the medial meniscus. Meniscal ossicles had been reported previously in tigers, lions, leopards, and jaguars [6, 7]. In tigers, medial meniscal ossification appears to be a normal anatomical variation that progressively develops following birth, and may serve as a model for the phylogenetic (developmental) theory of etiology [6]. In addition, the lateral and medial collateral ligaments were visualized in the dorsal and transverse planes. Other structures of femopatellar joint such as the patellar ligament and infrapatellar fat pad were both visualized in the sagittal and transverse planes, whereas the lateral and medial patellofemoral ligaments were visualized in the transverse planes. Similar observations of the anatomical structures of the stifle joint in all the three planes have been described in dogs [14–16] and horses [17].

The gross anatomical dissections used in this study contributed to a correct morphologic and topographic evaluation of the bony and soft tissue structures of the Bengal tiger stifle joint, and were a helpful tool for the identification of the MR images.

The use of MRI in captive tiger is currently limited because of its expense, availability, and the logistical problems of acquiring MR images in these animals, but its use in wildlife is justified in captive species because of the amount of diagnostic information obtained with little risk to the animal’s evaluated [12, 13]. This study used only one animal due to the difficulty to access to these species in Gran Canary Island. In the future would be interesting perform additional studies with higher number of animals to better evaluate all the anatomical structures of the stifle joint using high field MRI, as well as to establish a protocol for diagnosis of diseases of this area.

## Conclusions

In the present study, MRI has provided some clinically relevant anatomical details of the bony and soft tissues structures of Bengal tiger stifle joint.

## Abbreviations

MRI: Magnetic resonance imaging
MR: Magnetic resonance
SE: Spin-echo
GE: Gradient-echo
TE: Echo time
TR: Repetition time
TI: Inversion time
CT: Computed tomography
